# Description of *Aequorivita aurantiaca* sp. nov. Isolated from Coastal Sediment, and Comparative Genomic Analysis and Biogeographic Distribution of the Genus *Aequorivita*

**DOI:** 10.3390/microorganisms11102518

**Published:** 2023-10-09

**Authors:** Jun-Cheng Liu, Yu-Qi Ye, Xin-Yun Tan, Zong-Jun Du, Meng-Qi Ye

**Affiliations:** 1 SDU-ANU Joint Science College, Shandong University, Weihai 264209, China; liujuncheng2023@126.com; 2 Marine College, Shandong University, Weihai 264209, China; yeyuqi2023@163.com (Y.-Q.Y.); 202017638@mail.sdu.edu.cn (X.-Y.T.); duzongjun@sdu.edu.cn (Z.-J.D.); 3 State Key Laboratory of Microbial Technology, Shandong University, Qingdao 266237, China; 4 Weihai Research Institute of Industrial Technology, Shandong University, Weihai 264209, China; 5 Shenzhen Research Institute of Shandong University, Shenzhen 518057, China

**Keywords:** *Aequorivita aurantiaca* sp. nov., *Aequorivita*, polyphasic taxonomy, coastal sediment, comparative genomic analysis, biogeographic distribution

## Abstract

A novel Gram-stain-negative, facultatively anaerobic, and non-motile bacterial strain, designated SDUM287046^T^, was isolated from the coastal sediments of Jingzi Port of Weihai, China. Cells of strain SDUM287046^T^ were rod-shaped with widths of 0.4–0.5 μm and lengths of 0.7–1.4 μm and could produce flexirubin-type pigments. Optimum growth of strain SDUM287046^T^ occurred at 33–35 °C, pH 7.0, and with 2% (*w*/*v*) NaCl. Oxidase activity was negative, but catalase activity was positive. Phylogenetic analysis based on 16S rRNA gene sequence revealed that strain SDUM287046^T^ was most closely related to *Aequorivita aquimaris* D-24^T^ (98.3%). The main cellular fatty acids were iso-C_15:0_, anteiso-C_15:0_, iso-C_17:0_ 3–OH, and summed feature 9 (comprised of iso-C_17:1_ ω9c and/or C_16:0_ 10-methyl). The sole respiratory quinone was MK-6. The polar lipids consisted of phosphatidylethanolamine (PE), one aminolipid (AL), three unidentified glycolipids (GL), and three unidentified lipids (L). The DNA G + C content was 39.3 mol%. According to the integrated results of phylogenetic, physiological, biochemical, and chemotaxonomic characteristics, we propose that strain SDUM287046^T^ represents a novel species of the genus *Aequorivita*, for which the name *Aequorivita aurantiaca* sp. nov. is proposed. The type strain is SDUM287046^T^ (=KCTC 92754^T^ = MCCC 1H01418^T^). Comparative genomic analysis showed that the 16 *Aequorivita* species shared 1453 core genes and differed mainly in amino acid metabolism, cofactor metabolism, and vitamin metabolism. Biogeographic distribution analysis indicated that the marine environments were the primary habitat of *Aequorivita* bacteria.

## 1. Introduction

The marine environment, especially coastal sediment, is a very active ecosystem for material circulation and energy flow, which breeds various and abundant bacteria. However, a large number of uncultured bacteria in the ocean are difficult to culture under artificial conditions, which greatly limits the study of the ecological function and active products of marine bacteria. Therefore, it is a difficult and important task to achieve the isolation and culture of uncultured marine bacteria.

The genus *Aequorivita* was first proposed by Bowman and Nichols in 2002 as a member of the family *Flavobacteriaceae*, with *A. antarctica* being the type species [1], and was amended by Park et al. in 2009 [2]. At the time of writing, the genus *Aequorivita* consists of 18 validly published species according to the List of Prokaryotic Names with Standing in Nomenclature (LPSN, https://www.bacterio.net/species, accessed on 3 June 2023), including 7 species reclassified from the genus *Vitellibacter* to the genus *Aequorivita* [3,4,5]. Most members of the genus *Aequorivita* were isolated from marine environments such as seawater [1,6], marine sediment [7,8], cold seep [9], shallow water hydrothermal vent [10], seaweed [11], holothurian [12], and the intestinal tract of a squid [13], except for *A. sublithincola* (isolated from quartz stone subliths) [1] and *A. lutea* (isolated from estuarine sediment) [5]. The genus *Aequorivita* is widely distributed and is a potential group that can produce active compounds. In 2018, a study showed that *Aequorivita* sp. had antimicrobial and anthelmintic activity towards multidrug-resistant bacteria and the nematode *Caenorhabditis elegans* [14,15]. In addition, one strain of the genus *Aequorivita* was capable of producing esterase with polyethylene terephthalate (PET)-hydrolyzing activity, which suggested the potential ecological role of the genus *Aequorivita* in the decomposition of marine PET litter [16].

In this study, a rod-shaped, facultatively anaerobic strain was obtained from coastal sediments, and polyphasic taxonomic data suggested that the isolate can be classified as a representative of the novel species within the genus *Aequorivita*.

## 2. Materials and Methods

### 2.1. Bacterial Isolation and Cultivation

Strain SDUM287046^T^ was isolated from a coastal sediment sample that was collected from Jingzi Port in Weihai, China (122°7′38.80″ E, 37°33′57.60″ N) in November 2018. The sampling depth was about 5 m underwater. The temperature of the samples was 16 °C, the salinity was 40‰, and the pH was 8.0. Approximately 10 g of sediment was added to 90 mL of sterilized seawater and shaken fully. The mixed sample solution was diluted to 10^−2^ with sterilized seawater using the standard 10-fold dilution technique. Then the 100 μL dilution was spread on the “sandwich agar plate” designed in our laboratory as follows: The bottom layer was marine agar 2216 (MA; Becton Dickinson, Franklin Lakes, NJ, USA), on which the bacterium *Rhodovibrio salinarum* DSM 9154^T^ (Deutsche Sammlung von Mikroorganismen und Zellkulturen, Braunschweig, Germany) was cultured as a “helper” (the middle sandwich layer), and the top layer was the modified medium (per liter: 0.1 g yeast extract, 0.5 g peptone, and 40 g agar) prepared with artificial seawater (per liter deionized water: 3.2 g MgSO_4_, 2.2 g MgCl_2_, 1.2 g CaCl_2_, 0.7 g KCl, 0.2 g NaHCO_3_, and 30 g NaCl), as described previously [17]. After the incubation at 28 °C for one week, strain SDUM287046^T^ was isolated using plate streaking and subcultured serially on MA medium. Pure cultures were stored at –80 °C in a sterile 1% (*w*/*v*) saline solution supplemented with 20% (*v*/*v*) glycerol. The experimental strains *A. aquimaris* KCTC 42708^T^ and *A. antarctica* DSM 14231^T^ were obtained from the Korean Collection for Type Cultures (KCTC, Jeollabuk-do, Republic of Korea) and the Deutsche Sammlung von Mikroorganismen und Zellkulturen (DSMZ, Braunschweig, Germany), respectively, and were cultured under optimum conditions.

### 2.2. The 16S rRNA Gene Sequencing and Phylogenetic Analysis

Amplification of the 16S rRNA gene was performed using PCR technology with the primer pairs 27F and 1492R [18]. To obtain the nearly complete cloned 16S rRNA gene sequence of SDUM287046^T^, the clone operation was performed as described previously [19]. The purified PCR product was ligated into the pMD18-T vector (Takara Bio Inc., Dalian, China), and then the ligation product was transferred into *Escherichia coli* DH5a receptor cells. The 16S rRNA gene was sequenced by RuiBiotech (Qingdao, China), and alignment analysis was carried out by employing the EzBioCloud server (http://www.ezbiocloud.net/, accessed on 6 June 2023) and the NCBI database (https://blast.ncbi.nlm.nih.gov/Blast.cgi, accessed on 6 June 2023). The 16S rRNA gene phylogenetic trees were reconstructed with the neighbor-joining (NJ) [20], maximum-likelihood (ML) [21], and maximum-parsimony (ME) [22] algorithms employing the software MEGA X (version 10.2) [23]. The ML tree was reconstructed using the best-fit substitution model K2 + G + I. Bootstrap analysis was performed with 1000 replications to evaluate tree topologies.

### 2.3. Whole-Genome Sequencing, Genomic and Phylogenomic Analyses

Genomic DNA of strain SDUM287046^T^ was extracted using a bacteria genomic DNA kit (Takara Bio Inc., Dalian, China) and sequenced by Novogene Bioinformatics Technology Co., Ltd. (Beijing, China) on the NovaSeq 6000 platform (Illumina, San Diego, CA, USA). The raw reads were generated by the base-calling software CASAVA (version 1.8; Illumin, San Diego, CA, USA) and filtered using the data quality control software Fastp (version 0.23.0; HaploX Biotechnology Co., Ltd., Shenzhen, China). High-quality clean data were assembled using the software SOAPdenovo2 (version r242; BGI Genomics Co., Ltd., Shenzhen, China). The genomes of other relevant strains in this study were downloaded from the NCBI prokaryotic reference genome database.

The completeness and contamination values were estimated based on the method of lineage-specific CheckM (version 1.1.6) [24], and the complete 16S rRNA genes were extracted from the genome using the algorithm ContEst16S [25]. Genome annotation was performed using the NCBI Prokaryotic Genome Annotation Pipeline (PGAP) based on ab initio gene prediction algorithms and homology-based methods [26]. The analysis of secondary metabolite biosynthetic gene clusters in the strain SDUM287046^T^ genome was accomplished by the online antiSMASH server (version 7.0; https://antismash.secondarymetabolites.org/, accessed on 9 June 2023) [27].

Average nucleotide identity (ANI) and digital DNA–DNA hybridization (dDDH) values between strain SDUM287046^T^ and experimental strains were calculated using JSpeciesWS (https://jspecies.ribohost.com/jspeciesws/, accessed on 12 June 2023) [28] and the Genome-to-Genome Distance Calculator (version 3.0; http://ggdc.dsmz.de/ggdc.php, accessed on 12 June 2023) [29], respectively. The concatenated alignment sequences of 120 ubiquitous single-copy proteins were obtained by GTDB-Tk (version 1.3.0) [30], and the phylogenetic tree was reconstructed by FastTree [31] using JTT + CAT parameters and IQ-TREE [32] using the LG + F + I + G4 model with 1000 bootstrap replicates.

### 2.4. Comparative Genomic Analysis and Biogeographic Distribution of the Genus Aequorivita

Genome statistics of the strains within the genus *Aequorivita* used in this analysis are listed in Table S1. All genomes were predicted by the Prodigal tool [33], and the metabolic pathways were analyzed in detail employing KEGG’s BlastKOALA server (https://www.kegg.jp/blastkoala/, accessed on 3 June 2023) [34]. In order to estimate the genomic diversity and identify orthologous groups among the members of the genus *Aequorivita*, pan-genome analysis using the bacterial pan-genome analysis (BPGA) tool was performed with default parameters (50% amino acid sequence identity) [35].

To evaluate the global distribution and habitat preference of the genus *Aequorivita*, the analysis pipeline Microbe Atlas Project (MAP, https://microbeatlas.org/, accessed on 30 July 2023) was used with a 96% sequence similarity threshold. The quantification of the microbial abundances in the sequenced microbial communities was based on a closed reference ribosomal RNA analysis using MAPseq [36]. The use of a common reference enabled the direct comparison of microbial taxa abundance across samples and studies using different sequencing protocols. The analyzed sequencing data included amplicon, shotgun, and metatranscriptomic sequencing.

### 2.5. Morphology, Physiology, and Biochemistry

The physiological and biochemical features of strain SDUM287046^T^ were examined after incubation at 35 °C for 48 h on MA medium. Cell morphology and size were examined employing light microscopy (model E600; Nikon, Tokyo, Japan) and scanning electron microscopy (model Nova NanoSEM450; FEI, Portland, OR, USA). The Gram staining reactions were tested using crystal violet and safranin O stain solutions (Sangon Biotech Co., Ltd., Shanghai, China) according to the steps of the standard Gram reaction [37]. The presence of flexirubin-type pigments was determined using the alkaline method described previously (suspend the colonies in 20% KOH) [38,39]. The gliding motility test was carried out by preparing a cell suspension in seawater and then placing a drop on the marine 2216 medium solidified with 0.3% agar [40]. Growth range and optima of temperature were tested on MA at 0, 4, 20, 25, 28, 30, 33, 35, 37, 40, and 43 °C. Tolerance to NaCl was examined using the following medium (per liter: 1 g yeast extract and 5 g peptone), prepared with artificial seawater (per liter: 3.2 g MgSO_4_, 2.2 g MgCl_2_, 1.2 g CaCl_2_, 0.7 g KCl and 0.2 g NaHCO_3_) containing NaCl at concentrations from 0 to 10% (*w*/*v*, in 1% intervals). The pH range for growth was tested in marine broth 2216 (MB; Becton Dickinson, Franklin Lakes, NJ, USA) by adding different pH buffers at a concentration of 20 mM: MES (pH 5.5 and 6.0), PIPES (pH 6.5 and 7.0), HEPES (pH 7.5 and 8.0), Tricine (pH 8.5), and CAPSO (pH 9.0 and 9.5) (Sangon Biotech Co., Ltd., Shanghai, China). Bacterial growth monitoring was achieved with a spectrophotometer (model 900H; Perkin Elmer, Waltham, MA, USA) at 600 nm. Susceptibility to antibiotics was tested using the disc diffusion method described previously [41]. Anaerobic growth was examined after incubation for two weeks on MA in an anaerobic jar (10% H_2_, 10% CO_2_, and 80% N_2_).

Catalase and oxidase activities were assessed employing a 3% H_2_O_2_ solution and an oxidase test reagent (BioMérieux, Shanghai, China), respectively. Hydrolytic activity assays of starch (2%, *w*/*v*), alginate (2%, *w*/*v*), CM-cellulose (0.5%, *w*/*v*), casein (1% skimmed milk, *w*/*v*), and Tween (20, 40, 60, and 80, 1%, *w*/*v*) (Sangon Biotech Co., Ltd., Shanghai, China) were achieved following the methods described previously [42]. The API 50CH kits (BioMérieux, Shanghai, China) and Biolog GEN III MicroPlates were employed to test fermentative acid-producing activity and sole carbon source oxidation, respectively. Additional enzyme-producing activities were assessed using API ZYM and 20E kits (BioMérieux, Shanghai, China). All API and Biolog assays were performed simultaneously with the experimental strains according to the instructions, except that the NaCl concentration was adjusted to be optimal.

### 2.6. Chemotaxonomy

Comparative analyses of chemotaxonomic properties (fatty acids, isoprenoid quinone, and polar lipids) between strain SDUM287046^T^ and the experimental strains were carried out using cells harvested from MB medium at the late stage of the exponential growth phase. The culture temperature of SDUM287046^T^ was 35 °C, and the rotary shaker speed was about 200 r/min. The fatty acids were extracted by saponification, methylation, and extraction and then analyzed using a gas chromatograph (model 6890N; Agilent, Beijing, China) and the Sherlock Microbial Identification System (MIDI, version 6.1) [43]. Respiratory quinones were extracted as follows: Add 300 mg of freeze-dried pure cultures to the 40 mL mixed solution of chloroform and methanol (2:1, *v*/*v*) and stir in the dark for about 10 h; add 200 µL of acetone to dissolve after filtration and distillation operations; use silica-gel thin-layer chromatography (TLC) plates and chromatographic solution (n-hexane: ether = 85:15, *v*/*v*) to separate the quinones into different classes [44,45]. Respiratory quinones were identified using high-performance liquid chromatography (HPLC, model LC-20AT; Shimadzu, Shanghai, China). The extraction of polar lipids was achieved using a solution consisting of chloroform, methanol, and water (2.5:5:2, *v/v/v*), and the types of polar lipids were identified by the two-dimensional TLC method [46].

## 3. Results and Discussion

### 3.1. Phenotypic Properties

The colony of strain SDUM287046^T^ was circular, smooth, and orange-colored. Cells of strain SDUM287046^T^ were Gram-stain-negative and rod-shaped with widths of 0.4–0.5 μm and lengths of 0.7–1.4 μm (Figure S1). The orange-colored colonies turned orange-red in a 20% KOH solution, and the orange-red color reverted to orange after adding acid to remove the alkaline environment, which indicated that SDUM287046^T^ could produce flexirubin-type pigments. Growth was observed at pH 6.0–9.0 (optimum, 7.0), temperatures of 16–37 °C (optimum, 33–35 °C), and in the presence of 1–5% (*w*/*v*, optimum, 2%) NaCl.

Strain SDUM287046^T^ could hydrolyze Tweens 20, 40, and 60, but not cellulose, alginate, or agar. The activities of catalase, gelatinase, alkaline phosphatase, and acid phosphatase were positive, but the activities of urease, galactosidase, glucosidase, β-glucuronidase, cystine arylamidase, and lysine decarboxylase were negative, which was consistent with the experimental strains *A. aquimaris* KCTC 42708^T^ and *A. antarctica* DSM 14231^T^. The oxidations of the sole carbon source (Biolog) were positive for dextrin, d-cellobiose, gentiobiose, d-salicin, N-acetyl-d-glucosamine, sodium lactate, gelatin, glycyl-l-proline, l-glutamic acid, methyl pyruvate, Tween-40, and sodium butyrate, but negative for d-melibiose, fucose, d-arabitol, d-aspartic acid, l-pyroglutamic acid, pectin, mucic acid, d-saccharic acid, and malic acid. Acids were produced from d-ribose, d-fructose, l-sorbose, d-turanose, d-lyxose, d-tagatose, esculin ferric citrate, and potassium 5-ketogluconate, but not from erythritol, arabinose, xylose, d-adonitol, β-methyl-d-xylopyranoside, l-rhamnose, dulcitol, d-manitol, d-sorbitol, α-methyl-d-mannopyranoside, α-methyl-d-glucopyranoside, glycogen, and d-xylitol.

Strain SDUM287046^T^ was resistant to (μg per disc unless indicated) streptomycin (10), gentamycin (10), tobramycin (10), gentamycin (30), neomycin (30), and tetracycline (30), but sensitive to erythromycin (15), erythromycin (30), ampicillin (10), penicillin (10), chloramphenicol (30), rifampin (5), norfloxacin (30), cefotaxime sodium (30), clarithromycin (15), lincomycin (2), carbenicillin (100), and ceftriaxone (30). The differential characteristics between strain SDUM287046^T^ and the experimental strains are summarized in Table 1.

### 3.2. Chemotaxonomic Characteristics

The major cellular fatty acids of strain SDUM287046^T^ were iso-C_15:0_ and iso-C_17:0_ 3–OH, which were similar to *Aequorivita* species [2]. Furthermore, strain SDUM287046^T^ also contained anteiso-C_15:0_ and Summed Feature 9 (comprising iso-C_17:1_ ω9c and/or C_16:0_ 10-methyl) (Table S1). The sole respiratory quinone was MK-6, which was consistent with that observed for the related strains. The polar lipids consisted of phosphatidylethanolamine (PE), one aminolipid (AL), three unidentified glycolipids (GL), and three unidentified lipids (L) (Figure S2). The major polar lipids of strain SDUM287046^T^ were similar to those of experimental strains in that phosphatidylethanolamine and glycolipid were major components.

### 3.3. The 16S rRNA Gene Sequence and Phylogenetics

**Figure 1:**
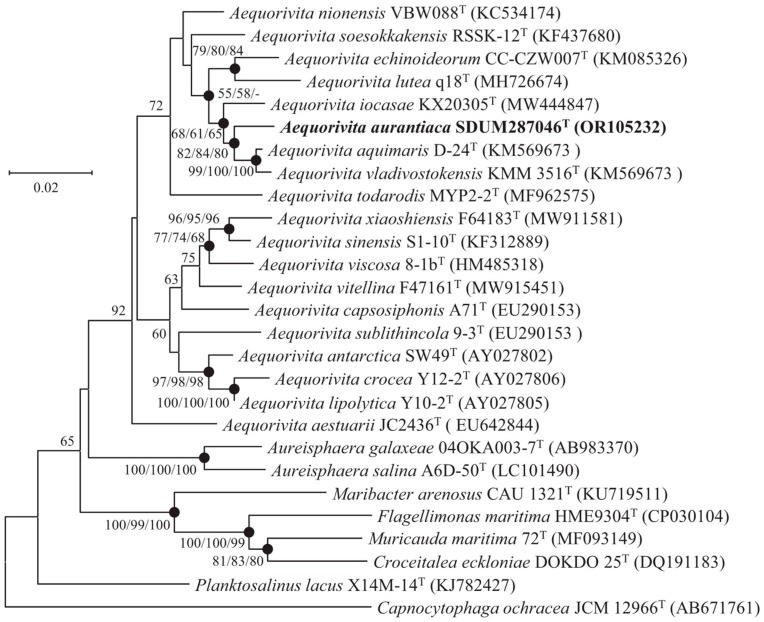
Neighbor-joining phylogenetic tree based on 16S rRNA gene sequences of strain SDUM287046^T^ and other closely related species. Filled circles indicate branches that were recovered with neighbor-joining, maximum-likelihood, and minimum-evolution methods. Bootstrap values above 50% (1000 replicates) are shown at branch nodes (NJ/ML/ME). *Capnocytophaga ochracea* JCM 12966^T^ was used as the outgroup. Bar: 0.02 substitutions per nucleotide position.

The 16S rRNA gene sequence of strain SDUM287046^T^ (1488 bp) was aligned with the EzBioCloud database, showing that the strain had 92.6–98.3% similarity values with members of the genus *Aequorivita* and shared the highest with *A. aquimaris* KCTC 42708^T^ (98.3%). Phylogenetic tree analysis based on 16S rRNA gene sequences showed that strain SDUM287046^T^ was clustered into the genus *Aequorivita*, which could be considered to represent a novel representative of the genus *Aequorivita* (Figure 1). The similar topologies of strain SDUM287046^T^ and related species were also obtained in the phylogenetic trees reconstructed with the ML and ME algorithms.

### 3.4. Genomic Features and Phylogenomics

The draft genome of strain SDUM287046^T^ had a total length of 3,093,921 bp with 53 scaffolds after assembly. The DNA G + C content was estimated to be 39.3 mol%. Genome completeness and contamination values were 98.9% and 0.5%, respectively. One 16S rRNA gene sequence (1524 bp) was detected from the genome, which has 100% similarity with the cloned 16S rRNA gene sequence obtained from amplification. According to the results of PGAP, the genome of strain SDUM287046^T^ contained 2878 genes, including 2825 protein-coding genes, 11 pseudogenes, and 42 RNA genes (3 rRNA, 35 tRNA, and 4 ncRNA). According to the results of antiSMASH, three secondary metabolite biosynthetic gene clusters encoding the types of aryl polyene, resorcinol, Type III polyketide synthase (PKS), and terpene were predicted in the genome of strain SDUM287046^T^. Among them, the gene cluster encoding aryl polyene and resorcinol types had the highest similarity value (75%) with the known flexirubin biosynthetic gene cluster from *Flavobacterium johnsoniae* UW101. The gene cluster encoding terpene type shared a 28% similarity value with the known carotenoid biosynthetic gene cluster from *Algoriphagus* sp. KK10202C.

**Figure 2:**
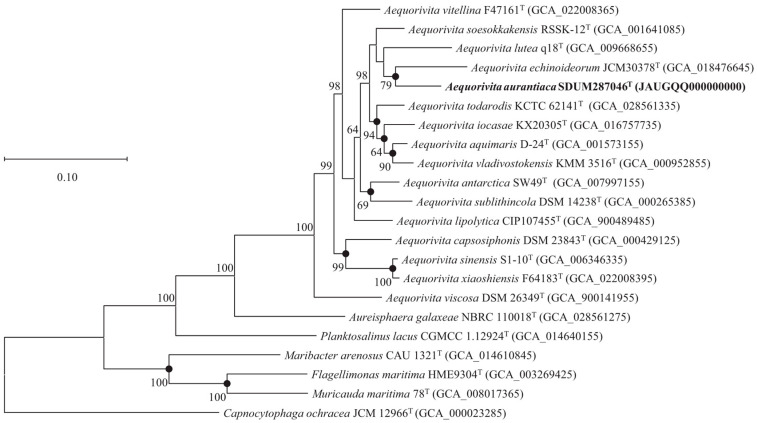
The FastTree is based on 120 ubiquitous single-copy proteins. Bootstrap values above 50% (1000 replicates) are shown at branch nodes. Filled circles indicate that the same topology is also obtained using the IQ-TREE algorithm. *Capnocytophaga ochracea* JCM 12966^T^ was used as the outgroup. Bar: 0.10 substitutions per nucleotide position.

The ANI and dDDH values between strain SDUM287046^T^ and *A. aquimaris* D-24^T^ (*A. antarctica* DSM 14231^T^) were 77.6% (78.3%) and 20.8% (21.5%), respectively, which were lower than the species delineation thresholds of 95–96% for ANI and 70% for dDDH [47,48]. The protein phylogenetic tree, showing the evolutionary relationships of strain SDUM287046^T^ and some related type strains, indicated the strain was affiliated with the genus *Aequorivita* (Figure 2), which was consistent with the result of 16S rRNA gene phylogenetic analysis.

### 3.5. Comparative Genomic Analysis of the Genus Aequorivita

**Figure 3:**
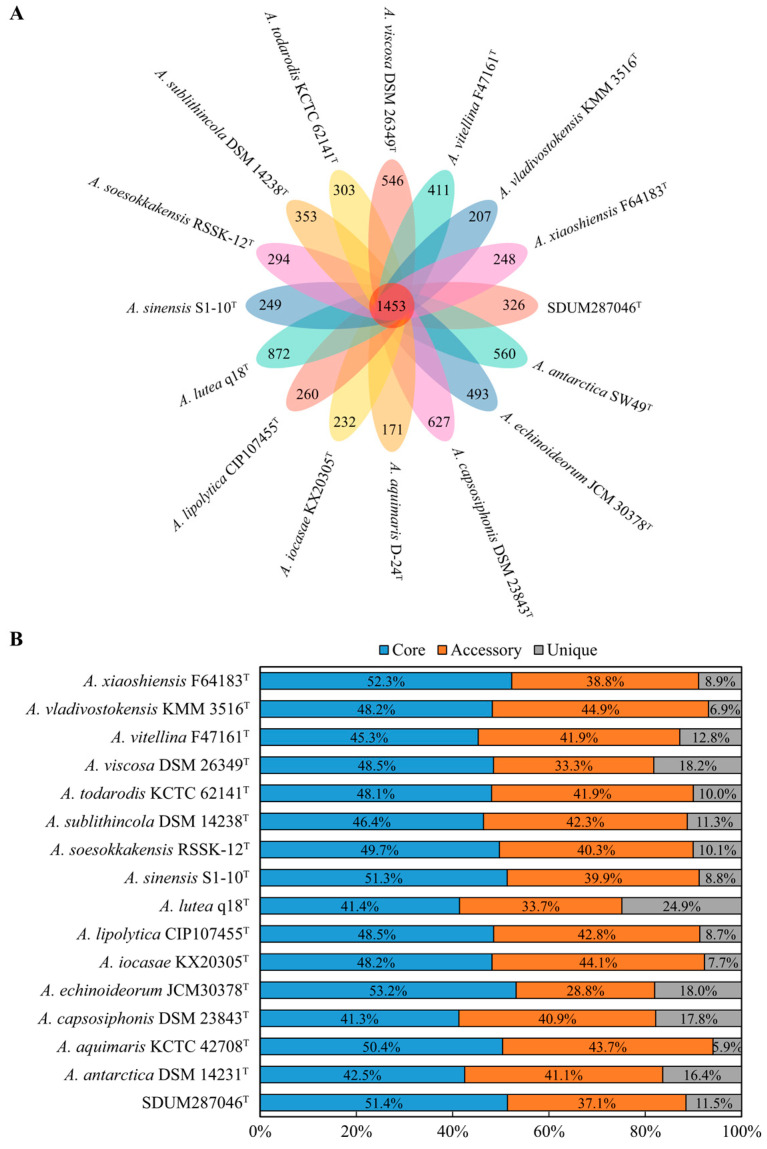
Pan-genome analysis of the genus *Aequorivita* (16 genomes). (**A**) Venn diagram displaying the numbers of core gene families and unique genes for each *Aequorivita* strain. (**B**) Percentage of core, accessory, and unique genes in each genome.

The size of all *Aequorivita* genomes, including strain SDUM287046^T^, ranged from 2,929,928 bp to 4,042,904 bp, and the GC contents ranged from 34.5% to 42.8% (Table S2). As presented in Figure 3, the pan-genome analysis based on orthologous groups of proteins revealed that 1453 core genes were shared by the 16 *Aequorivita* strains, which was about half of each genome. The percentage of accessory genes in each *Aequorivita* genome ranged from 28.8% to 44.9% and unique genes from 5.9% to 24.9%. Moreover, the analysis of KEGG distribution showed that the core genes were more involved in the metabolisms supporting basic life activities, such as nucleotide metabolism and translation, while unique genes were more distributed in carbohydrate metabolism, cell motility, cellular community, and membrane transport pathways (Figure S3), which might give the genus *Aequorivita* species metabolic diversity and flexibility [19].

**Figure 4:**
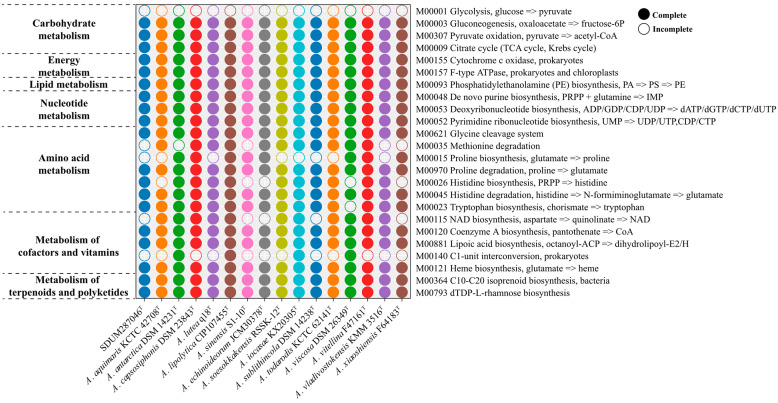
The metabolic module integrity of *Aequorivita* strains. The solid circles and hollow circles indicate that the metabolic pathways were complete and incomplete, respectively, as shown in the legend.

According to the KEGG annotation analysis (Figure 4), the TCA cycle pathway (M00009) and F-type ATPase (M00157) were complete in all *Aequorivita* genomes, while the glycolysis pathway (M00001) was not. Incomplete glycolysis pathways kept *Aequorivita* strains from fermenting with glucose as the sole carbon source in anoxic environments, which was consistent with the results of API 50CH kits. Considering that pyruvate deficiency due to glycolysis pathway incompleteness leads to a lack of acetyl-CoA from the pyruvate oxidation pathway, fatty acid oxidation and amino acid metabolism may be the important sources of acetyl-CoA for the *Aequorivita* strains. The phosphatidylethanolamine (PE) biosynthesis pathway (M00093) was annotated in all *Aequorivita* strains, which accorded with the result of the phenotypic experiment (Figure S2). The *Aequorivita* species were relatively conservative in energy metabolism, lipid metabolism, and nucleotide metabolism and differed mainly in amino acid metabolism, cofactor metabolism, and vitamin metabolism. For proline metabolism pathways, all 16 members of the genus *Aequorivita* had a complete proline degradation pathway (M00970), but only *A. antarctica* DSM 14231^T^, *A. capsosiphonis* DSM 23843^T^, *A. lipolytica* CIP107455^T^, *A. sinensis* S1-10^T^, *A. echinoideorum* JCM30378^T^, *A. viscosa* DSM 26349^T^, *A. vitellina* F47161^T^, and *A. xiaoshiensis* F64183^T^ had a complete proline biosynthesis pathway (M00015). Proline is an effective compatible solute that can resist the adverse effects of hypertonic and low-temperature environments on cells [49,50], and proline is involved in regulating the balance of reactive oxygen species, providing oxidative stress protection to cells [51]. However, excess proline was detrimental to cell growth, and intracellular proline must be present at appropriate levels [52]. Differences in proline metabolism may provide opportunities for the genus *Aequorivita* to adapt to various environmental conditions. The complete histidine degradation pathway (M00045) was found in all genomes, but the intact histidine biosynthesis pathway (M00026) was not annotated in *A. aquimaris* D-24^T^, *A. sinensis* S1-10^T^, *A. echinoideorum* JCM30378^T^, *A. viscosa* DSM 26349^T^, and *A. xiaoshiensis* F64183^T^.

### 3.6. Biogeographic Distribution of the Genus Aequorivita

**Figure 5:**
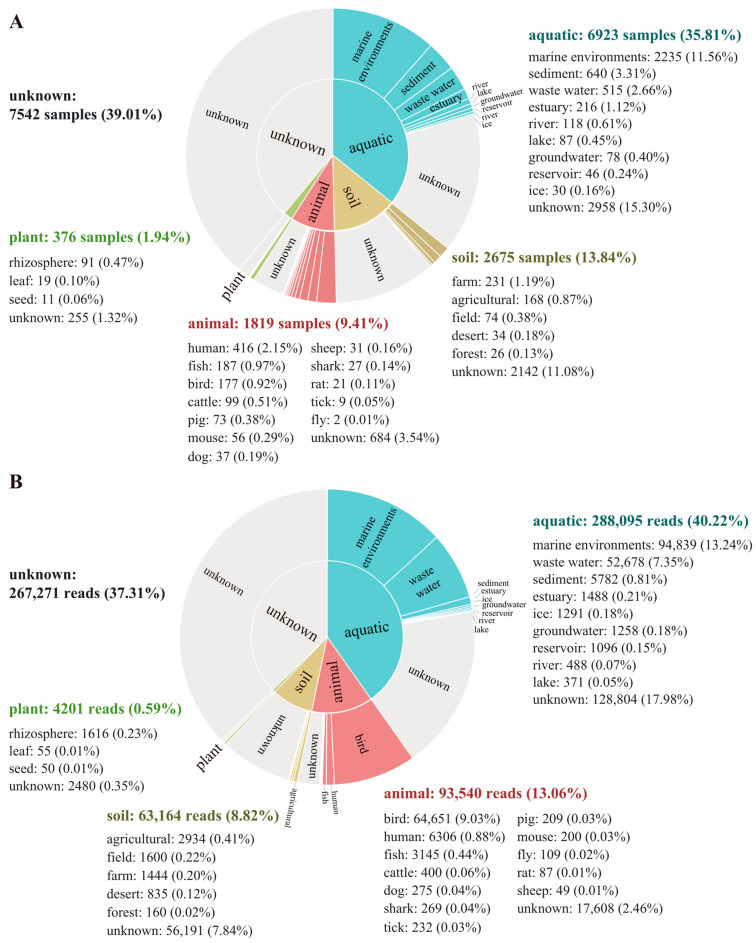
Biogeographic distribution analysis of the genus *Aequorivita* based on the Microbe Atlas Project (MAP) database and pipeline. (**A**) Number of samples containing the representative OTU sequence, per habitat and sub-habitat. (**B**) Number of sequencing reads mapping to the representative OTU sequence, per habitat and sub-habitat.

The global distribution of the genus *Aequorivita* was analyzed using the MAP database and pipeline, and the representative sequence was found in 19,335 samples from 3429 projects (details of the samples are summarized in Sheet S1). The results of biogeographic distribution analysis showed that members of the genus *Aequorivita* were widely distributed, including in aquatic environments, soil environments, animal environments, and plant environments. The *Aequorivita* bacteria were detected in 6923 aquatic samples (35.8%), but only 376 samples related plants (1.94%), and of the known aquatic environments, marine environments were the primary habitat (Figure 5A), which corresponded to the situation that most isolates were isolated from marine environments. The quantitative analysis of the database sequencing reads mapping to the representative OTU sequence showed the known habitat with the highest number of reads related to the genus *Aequorivita* was the marine environment (13.2%), followed by bird gut (9.0%) (Figure 5B).

### 3.7. Description of Aequorivita aurantiaca sp. nov.

*Aequorivita aurantiaca* (au.ran’ti.a’ca. N.L. fem. adj. *aurantiaca*, orange-colored, pertaining to the orange color of colonies).

Cells are rod-shaped and facultatively anaerobic with a positive Voges–Proskauer reaction. Nitrate cannot be reduced to nitrite, but flexirubin-type pigments are produced. The activities of arginine dihydrolase, tryptophan deaminase, gelatinase, esterase (C4), esterase lipase (C8), trypsin, and ɑ-chymotrypsin are positive, while those of cystine arylamidase, β-glucuronidase, galactosidase, glucosidase, N-acetyl-β-glucosaminidase, ɑ-mannosidase, and ɑ-fucosidase are negative. The major cellular fatty acids are iso-C_15:0_, anteiso-C_15:0_, iso-C_17:0_ 3–OH, and summed feature 9 (comprised iso-C_17:1_ ω9c and/or C_16:0_ 10-methyl). The sole respiratory quinone is MK-6. The polar lipids consist of phosphatidylethanolamine (PE), one aminolipid (AL), three unidentified glycolipids (GL), and three unidentified lipids (L). The DNA G + C content of the type strain is 39.3 mol%. The type strain is SDUM287046^T^ (=KCTC 92754^T^ = MCCC 1H01418^T^), which was isolated from coastal sediment samples of Jingzi Port in Weihai, China.

## Figures and Tables

**Table 1:** Differential characteristics between strain SDUM287046^T^ and the experimental strains.

Characteristics	1	2	3
**Isolation**	Coastal sediments	Seawater	Under-ice sea water
**Growth at 4 °C**	−	+	+
**NaCl range (%, *w*/*v*)**	1–5	0–9 *	0.5–10 ^†^
**Facultatively anaerobic**	+	−	−
**Enzyme activity:**			
Oxidase	−	+	−
Trypsin	+	w	+
ɑ-chymotrypsin	+	+	−
Gelatinase	+	+	w
**Hydrolysis of:**			
Tween 80	+	−	+
Starch	−	−	+
Casein	−	+	−
**Acid production from:**			
d-mannose	−	+	−
Potassium 2-ketogluconate	−	+	+
**Oxidation of:**			
d-maltose/d-trehalose	−	+	+
Gentiobiose	+	−	−
β-methyl-d-glucoside	+	−	−
N-acetyl-d-galactosamine	+	−	+
Inosine/Myo-inositol	−	−	+
l-alanine/l-serine	+	−	+
l-galactonic acid lacton	+	−	−
**DNA G + C content (mol %)**	39.3	40.6 *	38.5 ^†^

Strains: 1, SDUM287046^T^; 2, *A. aquimaris* KCTC 42708^T^; 3, *A. antarctica* DSM 14231^T^. All data were from this study, unless indicated otherwise. +—positive; −—negative; w—weakly positive. All strains were positive for the following characteristics: arginine dihydrolase, tryptophan deaminase, alkaline phosphatase, leucine arylamidase, valine arylamidase, acid phosphatase, naphthol-AS-BI-phosphohydrolase, d-ribose, d-fructose, d-sorbose, esculin ferric citrate, d-turanose, d-lyxose, d-tagatose, and potassium 5-ketogluconate. * Data from [6], ^†^ data from [1].

## Data Availability

The GenBank accession number of *Aequorivita aurantiaca* SDUM287046^T^ for the 16S rRNA gene sequence is OR105232, for the whole-genome assembly is JAUGQQ000000000.

## Supplementary Materials

The following supporting information can be downloaded at: https://www.mdpi.com/article/10.3390/microorganisms11102518/s1, Figure S1: The distribution of core genes, accessory genes, and unique genes to different metabolic pathways in the genus *Aequorivita*. Figure S2: Scanning electron micrograph of cells of strain SDUM287046^T^. Bar: 5 μm. Figure S3: Two-dimensional TLC plate image of the total polar lipids of strain SDUM287046^T^ (a), *A. antarctica* DSM 14231^T^ (b), and *A. aquimaris* KCTC 42708^T^ (c). PE, phosphatidylethanolamine; PGL, phosphoglycolipid; AL, unidentified aminolipid; GL, unidentified glycolipid; L, unidentified lipid. Table S1: Genomic dataset of the strains analyzed in the pan-genome analysis. Table S2: Cellular fatty acid composition (%) of the strain SDUM287046^T^ and experimental strains. Sheet S1: Detailed information of the 19,335 samples (from 3429 projects) for biogeographic distribution analysis.
