# Supplementary material for: Description of Aequorivita aurantiaca sp. nov. Isolated from Coastal Sediment, and Comparative Genomic Analysis and Biogeographic Distribution of the Genus Aequorivita
Source: Microorganisms. 2023 Oct 9;11(10):2518. doi: 10.3390/microorganisms11102518 (PMC10608841; doi:10.3390/microorganisms11102518)
Supplement: Supplementary file 1 [file microorganisms-11-02518-s001.zip › microorganisms-2560857-Supplementary Materials.pdf]

Supplementary Materials

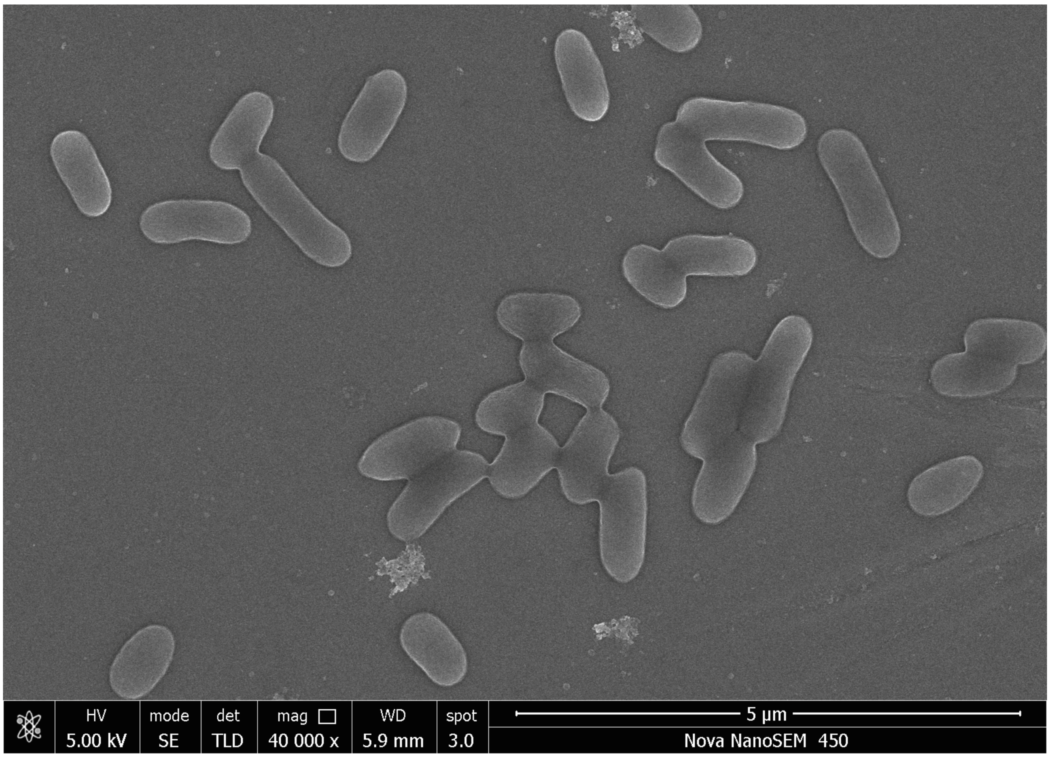

Figure S1. Scanning electron micrograph of cells of strain SDUM287046^T. Bar, 5 μm.

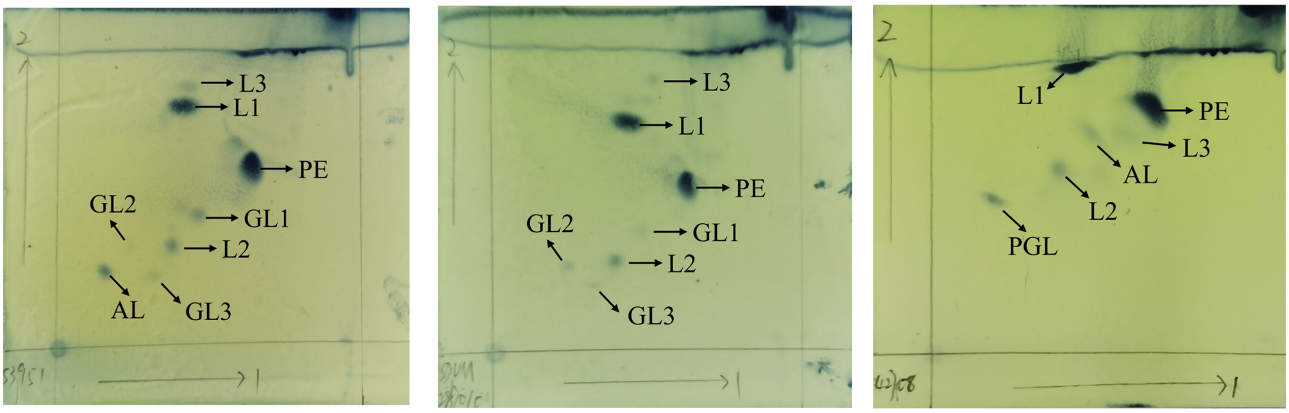

(a) (b) (c)

Figure S2. Two-dimensional TLC plate image of the total polar lipids of strain SDUM287046^T **(a)**, *A. antarctica* DSM 14231^T **(b)** and *A. aquimaris* KCTC 42708^T **(c)**. PE, phosphatidylethanolamine; PGL, phosphoglycolipid; AL, unidentified aminolipid; GL, unidentified glycolipid; L, unidentified lipid.

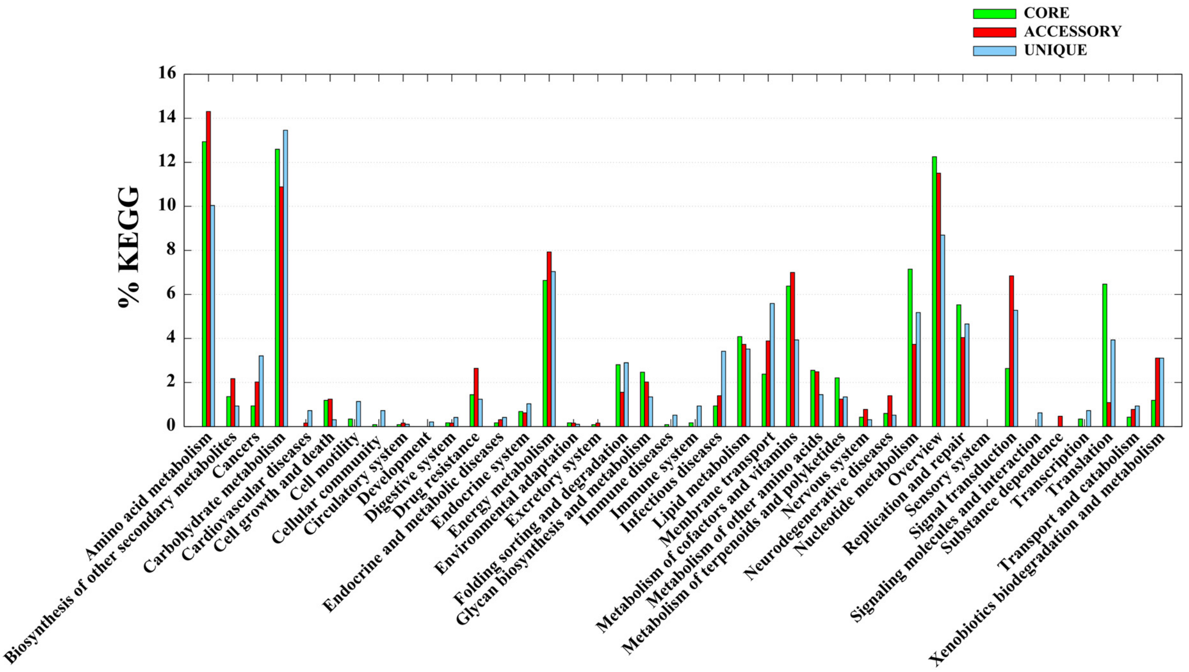

Figure S3. The distribution of core genes, accessory genes and unique genes to different metabolic pathways in the genus *Aequorivita*.

Table S1. Cellular fatty acids composition (%) of the strain SDUM287046^T and experimental strains.

**Fatty acid**	**1**	**2**	**3**
Saturated			
$C_{16:0}$	1.5	1.1	1.2
Branched			
iso-$C_{14:0}$	TR	2.2	TR
iso-$C_{15:0}$	**30.2**	**36.8**	**34.2**
anteiso-$C_{15:0}$	**17.2**	7.6	7.4
iso-$C_{15:1}$ G	2.5	7.2	8.1
iso-$C_{16:0}$	1.2	TR	TR
Hydroxy			
iso-$C_{15:0}$ 3–OH	1.8	3.1	3.8
$C_{15:0}$ 2–OH	1.4	TR	TR
iso-$C_{16:0}$ 3–OH	1.8	TR	1.4
iso-$C_{17:0}$ 3–OH	**14.9**	**20.0**	**21.6**
$C_{17:0}$ 2–OH	5.0	1.0	1.2
Summed Feature			
Summed Feature 3*	5.0	6.1	3.6
Summed Feature 9*	**11.6**	9.7	**11.1**

Strains: 1, SDUM287046^T; 2, *A. aquimaris* KCTC 42708^T; 3, *A. antarctica* DSM 14231^T. All data listed in the table are from this study. TR, trace (<1.0%); Fatty acids present at >10% are indicated in bold.

*Summed features are groups of two or three fatty acids that cannot be separated by GLC using the MIDI system. Summed feature 3 consists of $C_{16:1}$ ω7c and/or $C_{16:1}$ ω6c and summed feature 9 consists of iso-$C_{17:1}$ ω9c and/or $C_{16:0}$ 10-methyl.

Table S2. Genomic dataset of the strains analyzed in the pan-genome analysis.

**Strains**	**Genome size**	**GC content (%)**	**Accession number**
strain SDUM287046^T	3,093,921	39.3	JAUGQQ000000000
Aequorivita antarctica SW49^T	3,844,651	37.2	GCA_007997155
Aequorivita aquimaris D-24^T	3,147,268	40.0	GCA_001573155
Aequorivita capsosiphonis DSM 23843^T	4,042,904	36.9	GCA_000429125
Aequorivita echinoideorum JCM30378^T	2,929,928	38.7	GCA_018476645
Aequorivita iocasae KX20305^T	3,353,883	38.7	GCA_016757735
Aequorivita lipolytica CIP107455^T	3,305,581	37.7	GCA_900489485
Aequorivita lutea q18^T	3,369,026	42.8	GCA_009668655
Aequorivita sinensis S1-10^T	3,181,804	34.6	GCA_006346335
Aequorivita soesokkakensis RSSK-12^T	3,190,776	37.9	GCA_001641085
Aequorivita sublithincola DSM 14238^T	3,520,671	36.2	GCA_000265385
Aequorivita todarodis KCTC 62141^T	3,331,876	41.1	GCA_028561335
Aequorivita viscosa DSM 26349^T	3,526,892	36.6	GCA_900141955
Aequorivita vitellina F47161^T	3,633,834	37.4	GCA_022008365
Aequorivita vladivostokensis KMM 3516^T	3,269,452	40.8	GCA_000952855
Aequorivita xiaoshiensis F64183^T	3,066,048	34.5	GCA_022008395
